# Fostering reproducibility, reusability, and technology transfer in health informatics

**DOI:** 10.1016/j.isci.2021.102803

**Published:** 2021-07-01

**Authors:** Anne-Christin Hauschild, Lisa Eick, Joachim Wienbeck, Dominik Heider

**Affiliations:** 1 Department of Data Science in Biomedicine, Faculty of Mathematics & Computer Science, Philipps University of Marburg, Hans-Meerwein-Strasse 6, Marburg, 35032, Germany

**Keywords:** health informatics, Bioinformatics, software engineering, software robustness

## Abstract

Computational methods can transform healthcare. In particular, health informatics with artificial intelligence has shown tremendous potential when applied in various fields of medical research and has opened a new era for precision medicine. The development of reusable biomedical software for research or clinical practice is time-consuming and requires rigorous compliance with quality requirements as defined by international standards.

However, research projects rarely implement such measures, hindering smooth technology transfer into the research community or manufacturers as well as reproducibility and reusability.

Here, we present a guideline for quality management systems (QMS) for academic organizations incorporating the essential components while confining the requirements to an easily manageable effort. It provides a starting point to implement a QMS tailored to specific needs effortlessly and greatly facilitates technology transfer in a controlled manner, thereby supporting reproducibility and reusability.

Ultimately, the emerging standardized workflows can pave the way for an accelerated deployment in clinical practice.

## Graphical abstract

**Figure:**
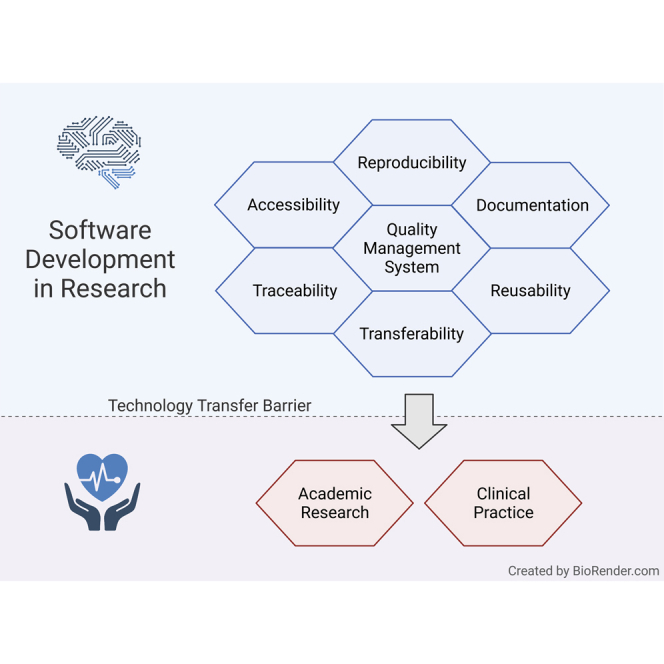

## Introduction

Computational approaches offer new opportunities to transform healthcare. Particularly modern artificial intelligence (AI) and machine learning (ML) techniques have shown substantial potential when applied in various fields of medical research and therefore opened up a new era for precision medicine (Hawgood et al., 2015). In the last decade, universities and other research organizations supported and encouraged by public funding agencies have allocated tremendous efforts to develop and enhance such predictive software models, algorithms, and “Systems as Medical Devices” (SaMD) for clinical research and application.

A manifold of studies have proven AI to be advantageous for disease diagnosis, prognosis, and disease monitoring (Center for Devices and Radiological Health, 2019; Fatima and Pasha, 2017). In cancer research, for instance, ML is used on omics data to gain deeper insights and understanding of the genetic and metabolic alterations that determine disease progression and enable tailored prognoses and monitoring (Batra et al., 2017; Hauschild et al., 2012; Jeanquartier et al., 2016; Wiwie et al., 2019). Additionally, computational models on clinical information are used to assess individualized health risks, for instance, to identify high-risk patients for sepsis in intensive care units (Calvert et al., 2019; Desautels et al., 2016), the analysis of longitudinal data for the early detection of heart failure (Chen et al., 2019), or applications in infectious diseases (Heider et al., 2014; Riemenschneider et al., 2016).

Once the outcome of this development is mature and robust to be published and used in routine treatment or diagnosis, there is a need to transfer this knowledge to other research groups and, ultimately, clinical practice. Therefore, a straightforward technology transfer to a manufacturer of medical devices would be beneficial (Brito et al., 2020; Riemenschneider et al., 2018).

### Challenges of scientific software development for health

While the knowledge and technologies to develop effective and efficacious AI-driven medical decision support systems exist, a manifold of pressing issues hinders further reusing in research and transfer to clinical practice. In software development projects in the industry, specialized developers typically work in large teams with considerably more resources that focus on usability and reusability (Guellec and de la Potterie, 2000; Mangulet al., 2019b). In contrast, academic teams often consist of small groups of researchers (e.g., graduate students and postdoctoral scholars) who are typically not trained software engineers and only have temporary contracts and frequent turnover. Thus, individuals often develop software on a one-person-one-project basis (Brito et al., 2020; Mangul et al., 2019b).

Moreover, funders and the academic hiring and promotion processes incentivize the pressure to publish and focus on “novelty” rather than software quality. Thus, it entices researchers to focus on theoretical aspects and *proof of concept* development (Brito et al., 2020; Mangul et al., 2019a). Therefore, most researchers implement software in a prototype-centered manner lacking quality checks such as systematic testing, which can be published quickly (Lee et al., 2021). However, these implementations often lack crucial qualities required for long-term reuse, such as documentation, usability, performance appropriate for real-life application, user-friendly interfaces, enabling reusability, or minimizing risk for potential users (Brito et al., 2020; Riemenschneider et al., 2018).

Recently, scientific journals such as GigaScience or Biostatistics have promoted **reproducibility** and **reusability** by mandating FAIR principles (i.e., Findability, Accessibility, Interoperability, and Reusability). FAIR establishes a guideline for scientific data management and documentation (Brito et al., 2020; Wilkinson et al., 2016).

However, as recently surveyed by Pinto et al. (2018), **documentation** of scientific software is one of the most significant “pain points” (Pinto et al., 2018). Journal publications are typically the primary source of documentation for scientific software and are quickly outdated by the agile software development style in academia (Mangul et al., 2019b). Ideally, documentation should be detailed enough so that a developer with no prior knowledge of the project should make productive use of the software and use it for further development without biases or limitations (Brito et al., 2020; Lee et al., 2021).

Another critical factor is **accessibility**. A lack of strict enforcement by journals, organizations, and funders has resulted in a loss of crucial data and software code (Brito et al., 2020). According to an extensive analysis by Mangul et al., almost 28% of all resources linked in publications were not accessible, indicating poor maintenance. Moreover, a large proportion of software tested by Mangul et al. was not usable due to non-installability or lack of portability. The main factors are storage locations outside of the journal’s directories or public versioning systems (Lee et al., 2021; Mangul et al., 2019b).

**Reproducibility** and **traceability** are two of the most important aspects of biomedical and health informatics (Coiera et al., 2018; Lee et al., 2021). The lack of publicly available and comprehensible source code undermines the auditing of published methods and results. Additionally, the traceability of changes via version control is critical for reproducibility and reuse of research and code that replicators get to use (Lee et al., 2021). These aspects ultimately undermine scientific rigor, transparency, and reproducibility (Brito et al., 2020). The previously described accessibility, documentation, portability, and reusability factors are essential to ensure reproducibility and underpin trust in the scientific record of scientific software (Lee et al., 2021).

Additionally, modern systems medicine approaches integrate all facets of private data such as electronic health records (EHR) (Shickel et al., 2017), laboratory results (Goecks et al., 2020), medical imaging (Anwar et al., 2018), omics resources such as the cancer genome atlas (TCGA), or the gene expression omnibus (Clough and Barrett, 2016; Hauschild et al., 2012; Jeanquartier et al., 2016; Tomczak et al., 2015), or pathway information (Batra et al., 2017; Jeanquartier et al., 2016; Wiwie et al., 2019). However, sensitive patient data that enables an association of confidential personal information to single individuals underlies strict regulations such as the European General Data Protection Regulation (GDPR) (Voigt and Von demBussche, 2017). Therefore, the exchange within and amongst institutes is perceived as insurmountable, posing a roadblock hampering significant data-based medical innovations.

### Quality management

The design and development of medical devices used in clinical practice, including software as a medical device, have to comply with regulations and standards that ensure the safety and quality of the medical devices worldwide (Maak and Wylie, 2016; World Health Organization and Others, 2011). These regulations include standards concerning quality management (QM), software development life cycle–including agile methods such as Scrum (Schwaber and Beedle, 2002) or Kanban (Anderson, 2010)–and risk management. Such measures are not yet a standard in academic research work (Mangul et al., 2019a, 2019b; Zamith and Gonçalves, 2018).

The challenges in academic development procedures, as described before, affect technology transfer as well as reproducibility and reusability amongst researchers as well as to the industry. It is usually restricted to transferring the intellectual method and know-how while discarding all created artifacts (e.g., documents for development planning and software code). In particular, manufacturers will need to understand the principles of the new method, train their staff members, and maintain design and development records when creating the final product. The record will include design documents, meeting minutes, and other documents produced during the development. This effort causes a significant overhead in the time and work they have to invest before the product is ready for the market (Sharma et al., 2013).

Introducing QMS by industry has proven to guarantee continuous high quality amongst projects and procedures within an organization. Our goal is to facilitate this experience in academic environments. Establishing an academia-tailored QMS for research organizations can address the described challenges of software quality, documentation, accessibility, traceability, and reproducibility and help software development in research organizations reach higher quality and standards. These quality standards are particularly critical for software that is intended to be used in the medical context. We can significantly reduce software transfer barriers amongst researchers in academia and industry if these standards are established from the start.

Moreover, it has the potential to facilitate and speed up the transfer to clinical practice and thereby improve medical diagnosis or treatment of patients rapidly. However, our recommendations are based on standards that are common for medical device development but do not have the intention to shortcut any steps of the regular medical device development; this will always have to be done according to the strict rules and regulations that apply. Other benefits of a QMS for medical device development are also beneficial for research organizations when developing such software. For example, clear responsibilities, new team members can be phased quickly, and everyone knows how their work contributes to project and organizational goals.

Requirements for QM are defined in ISO 9001 (Management and Devices, 2015), in ISO 13485 (Management and Devices, 2016), and the Code of Federal Regulation, CFR Title 21 (US FDA, 2017). However, while ISO 9001 is generic and can be used in different fields, ISO 13485 is specific for QM in medical device development. The main differences are that ISO 9001 requires the organization to demonstrate continual improvement. In contrast, ISO 13485 only requires the certified organization to verify that the QMS is effectively implemented and maintained. Additionally, ISO 9001 includes customer satisfaction, which is not relevant to ISO 13485. There are also some other differences in ISO 13485, e.g., that the promotion and awareness of regulatory requirements is a management responsibility, that risk assessment and management need to be carried out (according to ISO 14971), and some others. Unfortunately, ISO 13485 is most likely outside of what is feasible for research groups in universities and other research organizations.

### Our goal: Academia-tailored QMS

Until now, there is no guidance on how to establish an academia-tailored QMS for research organizations that balances between not having any QMS at all and a wholly formulated QMS that complies with official regulatory standards. Here, we present a synopsis of relevant requirements in such standards and adjust these toward software development requirements in academia. We aim to embrace the benefits and facilitate the technology transfer as much as possible while keeping the overhead for researchers and developers in a well manageable range. Thus, we tailor the content for use in universities or similar research organizations that focus on software in the medical setting.

However, the ideas presented here do not provide a shortcut when developing medical software. Any development of medical software must strictly follow the relevant regulations in the specific country or region. Therefore, our suggestions provide a starting point intended to be adapted to specific requirements as needed and we refer to it as MDx-ready (i.e., ready for transferring it into molecular diagnostics SaMD implementations). This flexibility is well in the mindset of most QMS: If there is a profound reason for the change, change it, but consider all relevant aspects and document the reasons and the changes.

## QMS for medical software research

A vital aspect to be clarified when setting up any QM is the scope of the activities. In principle, QM is set up on an organizational rather than a project level. One of the key benefits of a QMS is that it provides projects with guiding principles for the setup, documentation, and more. The organization sets up the QMS and the projects (and other procedures in the organization) use this QMS to ensure organization-wide quality and standards.

Universities and other research organizations often have a general QMS for the entire organization, primarily focusing on the quality of research and education. They cover topics such as hiring, waste disposal, publishing guidelines, and commitment to scientific standards. However, because of the range of specific subjects a QMS for medical software development software needs to cover, the standard university regulations are typically neither helpful nor sufficient. A university-wide QMS lacks particular requirements necessary in a customer-centered setting, such as for medical software development and defined in ISO 13485.

Traditionally, a company or organization defines a QMS to be applied in each project. In the academic setting, we recommend setting up a QMS, for instance, at the level of a research institute itself, an organizational unit such as a department, a subunit such as a research group, or agile organizations such as scientific consortia. For clarity, in this manuscript, we will use the general terms ‘organization’, ‘institute’, and ‘unit’ interchangeably. Which level is most appropriate may differ on a case-to-case basis. However, it is vital to make a dedicated decision before starting to set up such quality guidelines. To give a practical demonstration of an academic QMS implementation, we provide an example on the level of a research organization in supplemental informationS1.

### Content of the QM system

**Figure 1:**
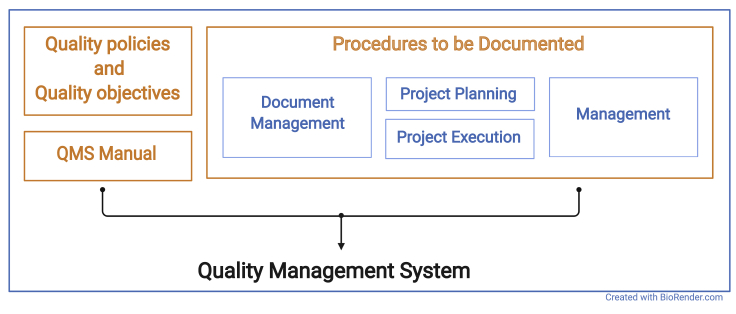
Main components and organization of a quality management system

The QMS of an organization or research unit defines procedures and structures that are implemented in the organization to be seen and understood by everyone. It describes the basic structure of an organizational unit and defines the procedures that are implemented in the organization. Procedures in this context may be administrational processes, such as the employee training process, or technical procedures, such as storage and archiving processes. The QM system documents inputs, outputs, responsibilities, and activities for each procedure (see Figure 1).

For an organization that works on research projects, the QMS should guide the execution of projects. In particular, it includes the basic structure, documentation, technical setup, responsibilities, and roles within projects. The guidance and policies of an organizational QMS ensure a controlled and defined project execution.

However, in a research environment, a well-designed QMS should be a guide, not a corset. Thus, the QMS should allow a significant degree of freedom to deviate from the recommended processes. Deviations are required to be documented, including a detailed justification and description of the alternative path chosen. It allows adaptations to different conditions while maintaining a solid level of traceability and reproducibility. Moreover, procedures necessary for a qualitative implementation and documentation that persons involved in a project commit to need to be described. Therefore, the QMS addresses all personnel involved in a project. Ultimately, by participating in the project, they commit themselves to comply with the procedures and regulations defined in the QMS.

### Quality policies and quality objectives

International standards for QMS for medical devices, such as the ISO 13485, require documenting an organization’s quality policy and quality objectives on the highest level. Clear quality policies and quality objectives will help understand what drives the organization and how it intends to reach its quality goals (Management and Devices, 2016).

A quality policy describes the high-level philosophy of the organization concerning quality. A quality objective is a measurable goal that is derived from quality policies. They describe what distinguishes an organization from other organizations and what drives the organization on a high level. Personnel must be aware of the relevance and importance of their activities and how they contribute. The quality policies condense the overall goals, allowing staff members to align their work with these overarching goals.

Moreover, a research organization implementing a limited QMS should declare in the quality policy to what extent the execution of the QMS is mandatory (policy) for all projects and how it intends to enforce this decision (objective). In contrast to manufacturers of medical device software, a full QMS implementation and execution is not mandatory for research organizations in this article’s scope. However, they should deliberately choose whether and to what extent they use a QMS. Supplemental informationS1.1 gives an example for a “Quality policy and quality objectives” description.

### Quality manual

The quality manual documents the core of the QMS. It defines the scope of the QMS and describes procedures and structures in the organization. We can consider it as the “instruction handbook” of the QMS. Ideally, it should be possible to hand a copy of the quality manual to a new employee in the organization, enabling that person to work according to the QMS (in reality, this is typically not possible due to the size and complexity of a QMS).

Independent of the complexity and intended environment a QMS is created for, it should always contain a quality manual. The document comprises, for instance, information on:

• Scope of the QMS (For whom is it relevant? Which procedures are covered? etc.)
• Structure of the QMS (One document? Multiple documents? How are multiple documents organized? etc.)
• How and where are the procedures of the QMS stored, maintained, and archived

Typically, a quality manual does not contain all the descriptions of all covered procedures, but includes references to other documents containing these descriptions. This structure makes it much easier to find and read the description of specific procedures. Also, it allows updating the definition of parts of the QMS rather than the entire QMS at once. The “Quality manual”” of the example can be found in supplemental informationS1.2.

## Procedures to be documented

This article aims to give guidance on what subset of a QMS is helpful to implement to facilitate technology transfer from research to a manufacturer of medical devices as well as for supporting reproducibility and reusability. So far, we have discussed the general aspects of a QMS, the following sections list, and briefly described which procedures we consider to be most important in this context. Established standards for QMS require a significantly more extensive set of procedures, and there are always good arguments for including or excluding a particular set of procedures (Management & Devices, 2015, 2016). We consider our recommendation as a starting point that can (MDx-ready), and should, be adapted if there is any good reason to do so.

### Document management

One of the most important things to aid the technology transfer to move forward from research to an industrial, full-blown medical product is documentation. It helps tremendously to have reliable, well-defined, structured, and (within the given possibilities) complete documentation.

The most fundamental aspect of document management is to define where and how documents are stored and managed. Different document storage systems are suitable; for instance, a file system, in a source code management system, such as git or subversion (Collins-Sussman, 2002; Spinellis, 2012), in a content management system, or a dedicated document management system. If all projects separately decide how to store documents, managing and retrieving documents on an organizational level will be much more difficult. This is especially true considering the fast turnover of employees typical in research-oriented groups. Therefore, it is advisable to use the same document storage for all documents relevant to the organization and document it in the QMS. The documents that make up the QMS should be stored accordingly. Thus, the decision on a storage system should precede the development of the QMS.

Additional items that this part of a QMS should cover are, for instance:

• How are documents identified consistently and unambiguously?
• How can users access and modify documents?
• How will previous versions of a document be available?
• Is any approval needed when creating/storing a new document or a new version?
• If permission is required, who needs to approve, and how is this done?
• How can a user be sure to have the current version of a document?
• Will documents be discarded at any time?

The set of procedures that cover these topics and others relevant to handling documents determines the document management section of the QMS. This section may also include the choice of a specific format or software for the work with documents.

We can distinguish between “documents” and “records”, where “documents” describe what is planned and “records” describe what happened. Documented procedures may support changes and updates, while records should never be altered after they were stored. For the sake of simplicity, we do not distinguish the two in the scope of this article. Supplemental informationS1.3 describes the “Procedures to be documented” for the example QMS.

### Project planning

Having some project planning is tremendously helpful, even in a research-oriented organization. It not only dramatically increases the likelihood of successful project completion, but it also makes it much easier to understand what happened in a project and why it happened— which in turn supports the technology transfer significantly.

Therefore, we propose to define a set of procedures on how to handle project planning as part of a QMS proposal. However, not every tiny project has to implement comprehensive project planning. It only clarifies when to do which kind of planning and sets guidelines for the implementation.

Essential elements to be covered here are for instance:

• A procedure to systematically choose which parts of the QMS will be applied to the project (might be a limited subset for small projects)
• A procedure that requires projects to check if everyone has the same understanding of the project goals
• A procedure that requires projects to define the roles and responsibilities within the project
• A set of documents that needs to be created for each project
• A special focus lies on risk assessment and management (according to ISO 14971)

An exemplified “Project planning” is given in supplemental informationS1.4.

### Project execution

Every project is subject to specific circumstances, boundary conditions, internal and external requirements, and so on, leading to variability in execution. While this may or may not be beneficial for each project, it usually makes technology transfer, reproducibility, and reusability much more complicated; as part of the transfer, all of the specific setups for the project need to be understood. Having some level of standardization for all projects in an organization and having this standardization documented consistently will make the transfer, reproducibility, and reusability much more manageable. Besides this, it also provides many benefits that may not be visible to the individual projects at the time of start–having a higher chance of avoiding pitfalls using experience from previous similar setups is one of these. Therefore, we recommend defining procedures for project execution as part of our QMS for research organizations.

More specifically, we recommend at least defining procedures for the following:

• Requirements management. Each project should at least spend thoughts on which inputs to consider for requirements, prioritize requirements, document requirements, and handle changes in requirements. A set of procedures that directs projects on how to spend these thoughts in a more formalized way should be part of the QMS.
• Definition of the development environment and tools: It is probably not helpful to have all projects use the same development environment and set of tools. But it is beneficial to require the projects to decide what to use in a structured way and document this decision. Include the procedures on how to make the decision and what to document in the QMS.
• Development process: As before, the most critical point is that selecting a specific development process (e.g., Scrum, Kanban, or Waterfall) is done consciously and that the reasons for the decision are documented.
• Completion of the project: It must be clear when a project is completed. Again, this may depend strongly on the individual project. Some are complete after successful testing, others simply when time is over. The important thing is that everyone has the same understanding of when this is the case. Therefore, there should be a procedure that requires each project to define the criteria for completion and document this decision.
• Documentation: The level of documentation created during the development (code comments? design document? test documentation? etc.) may be subject to different needs. However, it is good to have firm guidelines here, as documentation is typically an unpopular and often neglected activity.

The example description of the “Project execution” can be found in supplemental informationS1.2.

### Management

The success and quality of a venture or project primarily relies on the competencies of the contributing scientific and administrative personnel and their access to resources. Therefore, it is inevitable to provide them with the necessary know-how and competencies through appropriate education, training, skills, and experience processes. To ensure the consistent quality of all projects within an organization, the corresponding QMS should document such processes.

More specifically, we recommend at least defining procedures for the following:

• QMS training: The effort put into establishing a QMS is useless unless the staff members involved in the projects and the administration is trained in QMS. It is essential that every person is aware of the QM system, knows where to find the QMS documents associated with their work, and knows how to act accordingly. Therefore, it should be documented how the management will ensure appropriate QMS training for new employees and initiate regular update training for all personnel when the QMS is changed. Moreover, it is essential to determine who is responsible for ensuring that every employee has the required QMS competencies and how training is implemented. The training will ensure that project teams are implementing a QMS in practice. However, while according to QM system standards like ISO 13485, such training processes require meticulous documentation of participation for each employee, we suggest that this is not mandatory in the academic environment (Management and Devices, 2016).
• Qualification of personnel: The management is responsible for ensuring that all staff members are qualified for their responsibilities. The process may include hiring staff members according to the requirements of the project and providing necessary training. The QMS should define corresponding procedures and responsibilities.

Supplemental informationS1.6 describes the “Management” document of the example QMS.

## Discussion

Computational and data science approaches, such as machine learning or artificial intelligence, have opened a new era in health care and precision medicine such as diagnostics, prognostics, and monitoring. However, the development of such biomedical and health algorithms is very time-consuming. Moreover, transferring the knowledge and technology to clinical practice requires implementing all quality requirements defined in international standards.

Here, we discussed the difficulties of academic technology transfer, reproducibility, and reusability within research and from research to commercial manufacturing with a particular focus on software that is intended to be used in a biomedical and clinical context. Therefore, the well-described challenges of documentation, traceability, transparency, accessibility, replicability, and reusability affecting software quality need addressing (Brito et al., 2020; Lee et al., 2021; Mangul et al., 2019a).

To the best of our knowledge, we propose the first guidelines establishing an academia-tailored QMS for research organizations and units, which can significantly facilitate reproducibility and reusability of scientific software and speed up technology transfer in a controlled and predictable way. Only a few research organizations have started to implement such processes (Sapunar et al., 2016) yet. The developed guideline intends not to simplify the certification process but to support the manufacturer with gathering all required documentation for the certification process from the academic partners (MDx-ready). While a regulation-conform QMS is not feasible for most research organizations, we proposed a checklist of selected elements of a QMS that will significantly benefit software quality and reusability while keeping the effort in a range that most organizations can easily handle. Thus, our proposal focuses on document management, project planning, execution, and surrounding administrative procedures. However, depending on an organization’s specific needs, the set of elements in the QMS may need adjustment and differ from our suggestions.

Nevertheless, a commitment that a QMS is implemented and that elements are deliberately chosen to fit the organization is already a key factor in improving reproducibility, reusability, and facilitating technology transfer. In particular, community-wide adoption of such best practices for reproducibility would be critical to exploit the full potential of agile software development in the biomedical and life sciences (Brito et al., 2020). Ultimately, to break the circle of constantly reinventing the wheel in academic software development, the reuse of scientific software is inevitable (Lee et al., 2021). Our proposal provides a starting point for this, lowering the hurdle for research organizations to set up quality management.

## Limitations of study

This article presents a guideline for QMS for academic organizations incorporating only the essential components while confining the requirements to an easily manageable effort. However, direct implementation of the entire ISO 13485 is most likely outside of what is feasible in universities and other organizations. The ideas presented here do not provide a shortcut when developing medical software. Any development of medical software must strictly follow the relevant regulations in the specific country or region. Therefore, our suggestions provide a starting point intended to be adapted to specific requirements as needed. We refer to it as MDx-ready, which we define as ready for transferring it into molecular diagnostics SaMD implementations.

### Methods

All methods can be found in the accompanying transparent methods supplemental file.

## Footnotes

Supplemental information can be found online at https://doi.org/10.1016/j.isci.2021.102803.
