# Supplementary material for: Fostering reproducibility, reusability, and technology transfer in health informatics
Source: iScience. 2021 Jul 1;24(7):102803. doi: 10.1016/j.isci.2021.102803 (PMC8282945; doi:10.1016/j.isci.2021.102803)
Supplement: Document S1. Figure S1, Tables S1–S4, and Methods [file mmc1.pdf]

iScience, Volume *24*

Supplemental information

Fostering reproducibility, reusability, and technology transfer in health informatics

Anne-Christin Hauschild, Lisa Eick, Joachim Wienbeck, and Dominik Heider

Supplementary Material

Table of Content

A practical proposal for the implementation of a QMS 2

Quality policies, objectives and manual 3
Quality policies 3
Quality objectives 3

Quality manual 4

Document management 4
General requirements 4
Procedure DM-1: assignment 4
Procedure DM-2: documentation platform 5
Procedure DM-3: Documentation lifecycle 5
Procedure DM-4: Documentation change 5
Procedure DM-5: Meeting minutes 6
Procedure DM-6: Standard Operating Procedure documentation 6
Concrete example 7
Procedure DM-1: assignment 7
Procedure DM-2a: documentation platform 7
Procedure DM-2b: documentation platform (Code) 7
Procedure DM-3: Documentation lifecycle 8
Procedure DM-4: Documentation change 8
Procedure DM-5: Meeting minutes 8
Procedure DM-6: Standard Operating Procedure documentation 9

Project planning 10
Procedure PP-1: assignment 10
Procedure PP-2: prepare document storage 11
Procedure PP-3: list of documents 11

Project execution 12
Procedure PE-1: create a software development plan 12

Management 13
Procedure MA-1: assign quality management responsibility 13
Procedure MA-2: implement training for new staff members 13
Procedure MA-3: implement one department meeting each semester with QM update 13
Procedure MA-4: annual QM review 14

References 14

A practical proposal for the implementation of a QMS

The concepts of QMS systems are hard to grasp on a theoretical level. Therefore, we will present in the following a practical example of a QMS implementation in academia.

The Institute for Diagnostic Image-processing in Radiology (IDIR) is a fictional organization for the development of medical software focussing on the processing and interpretation of medical imaging technologies such as Computer Tomography. The size has a strong impact on what is feasible for an organization. We assume a size of roughly 100 employees within IDIR. To ensure structured processes and quality throughout the institute, we intend to set up an institution-wide QMS for IDIR. A well-defined QMS will improve reproducibility, reusability, and transfer. This example will give an idea of how a QMS for a real-world research organization could be implemented. Note that it is beneficial to define a QMS on an organizational level which will define more general aspects and processes. If such an organizational QMS does not exist, smaller organizational units can implement a QMS which will focus on specific aspects within the unit. In the following, we will give examples for information that could be included for the different sections of a potential QMS for the IDIR, see Figure S1 for an overview of the QMS components for IDIR.

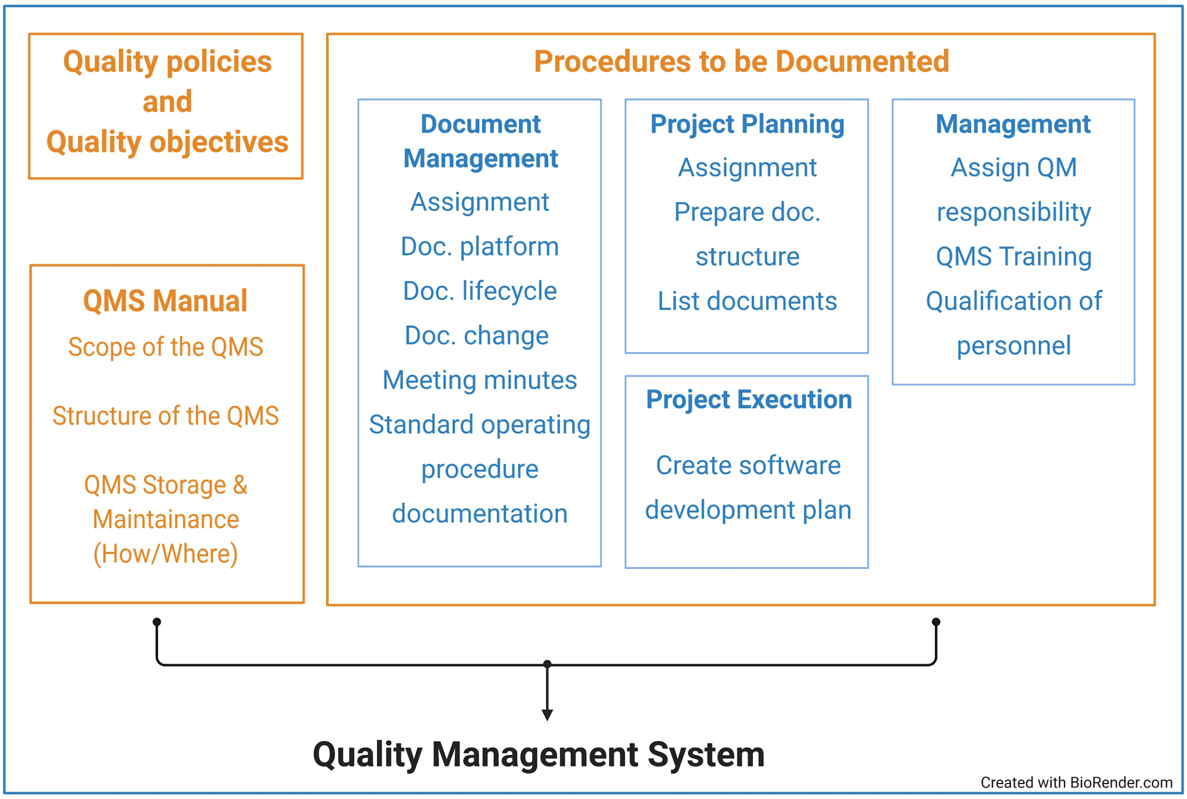

Figure S1. This figure shows the building blocks for the example quality management system of the IDIR institute.

1. Quality policies, objectives, and manual

An integral part of a QMS system is the definition of quality policies and objectives of the organization. While quality policies describe the high-level aims and attitude of the organization, quality objectives will define measurable goals that are derived from these policies.

1.1. Quality policies

The IDIR defines the quality policies in the following way: "Our institute is striving to **revolutionize the way we utilize medical imaging for information mining** with a focus on medical applications. Our efforts are therefore tailored to the processing and interpretation of imaging generated by technologies such as computer tomography (CT) or positron emission tomography - CT. Moreover, we aim to ensure that all **advancements will be publicly available to benefit society** and to improve reproducibility and reusability. Major objectives are the combination of this information with computational and artificial intelligence to optimize clinical diagnostics and therapy optimization for various diseases."

1.2. Quality objectives

Consequential the IDIR dedicates all its efforts to the following objectives:

- We aim to develop algorithms that enable the integration and analysis of medical images (as specified previously) and regularly publish open-source software packages and medical applications that support various aspects of interactive data interpretation for academic and medical research.
- We aim to build computational models that utilize medical imaging to optimize clinical diagnostics for tumors of, for instance, internal and external organs. Moreover, we will integrate these models into user-friendly software tools and applications to make them publicly available to support medical decisions.
- An integral objective of this institute is to facilitate a seamless knowledge and technology transfer to industrial partners for commercial release.
- Therefore, the IDIR commits to implementing the academically tailored QMS as described in the following. This document is accessible to all organizational groups and employees in the IDIR and it is mandatory to adhere to the described standards and recommendations. Exceptions must have a reasonable explanation and require a written justification.

2. Quality manual

As described before, the Quality manual is the central component of the QMS. It defines its scope and describes key procedures and structures in the organization.

For the IDIR, the QMS defines standard procedures and processes such as documentation and responsibilities that are relevant for all groups and employees in the institute. In particular, projects that contribute to the above-described objectives are encouraged to implement the QMS in their work.

The QMS of the IDIR is described in a set of documents highlighting different aspects and processes within the QMS. Central document management describes procedures involving various aspects of documentation and management thereof. For instance, the clarification of responsibilities (DM-1), the technical and organizational aspects of document storage (DM-2), document lifecycle from creation to disposal (DM-3), and changes affecting other documents and processes (DM-4). Moreover, it should comprise meeting minutes (DM-5) and generally defines the standard operating procedure for documentation (DM-6). Additionally, the QMS should include several documents for project planning and execution. For example, standardized processes could describe the assignment of responsibilities within a project (PP-1), technical aspects of document storage (PP-2), and a list of documents created for a project (PP-3), as well as a procedure for a software development plan (PE-1). Finally, procedures for different aspects of management can be determined, such as assigning responsibility for the QM (MA-1), the implementation of training (MA-2), organization of regular QM update meetings (MA-3), and the annual QM review (MA-4).

Moreover, the quality manual will give a detailed description of the location, maintenance, and lifecycle of the different documents within the QMS.

3. Document management

The following information is documented and available to every employee in the local intranet of the IDIR.

3.1. General requirements

Procedure DM-1: Assignment

The document management process is a vital part of the QMS and is therefore described in more detail below.

The first step is to appoint a person responsible for documentation, the documentation representative, to ensure that responsibilities for the implementation

of the document management procedures are clear. One of the tasks of this person is to take care that the following setup of the QMS is appropriately documented, e.g., minutes are written for meetings. Furthermore, the range of tasks of the documentation representative should be determined depending on the institute and its objectives and be appropriately assigned.

Procedure DM-2: Documentation platform

A documentation platform is needed that contains several functions:

- The most important thing is that the documents are not lost. Even documents marked as deleted must be retrievable at any time. This is of great importance to be able to track the entire work process.
- This is partly solved with the second property that such a platform should have versioning. This ensures that old versions of documents are not lost so that it is possible to track the progress of the project. Good versioning can also solve the problem of documents that need to be deleted. The latest version is marked as deleted and the document and its versions can still be found.
- The third characteristic of a good documentation platform is that everyone who reads and edits the documents also has the necessary access. However, it is equally important that documents cannot be changed by everyone but only by authorized employees.
- The last characteristic is that a good platform should have a system that allows you to mark whether employees have read documents. This is to make sure that everyone has read the important documents needed for working on projects of this instance.

Procedure DM-3: Documentation lifecycle

Furthermore, the lifecycle of the documentation should be defined. This typically consists of generating, editing, distribution, use, archiving, and disposal. It should be precisely defined how the steps should be carried out to ensure that the documentation is uniform and consistent.

Procedure DM-4: Documentation change

The process of document changes should also be recorded in the QMS. This is because important steps must also be followed here. When making changes in the documents, it should be taken into account that changes also may affect other documents and processes. For this purpose, all objects that could be affected by the change should be identified first and whether they also need to be adjusted.

After this elaboration, the changes must be confirmed by persons with permission.
Finally, the changes are implemented.

Procedure DM-5: Meeting minutes

In most cases, each employee is responsible for documenting their work. However, in meetings where many people are working on a project, it can happen that the responsibility is not clear. It is therefore advisable to create a process for recording the meeting.

Procedure DM-6: Standard operating procedure documentation

Now that the basic structures are in place, the emphasis is on uniform documentation where possible. Since requirements for documents can change quickly and frequently, it is not recommended to integrate them explicitly into the QMS. For this, the possibility exists to define specific details in "SOP" (Standard Operating Procedure) documents, which are referenced by the QMS, but not part of the QMS. This allows the documents to be modified without having to modify the QMS itself. The SOP documents should not define procedures or organizational structures, but rather be used for document templates, etc.

All physical (paper) documents and records will be scanned and stored in the file system unless it is physically not possible. Subsequently, paper documents may be discarded, unless this would result in a loss of information (e.g., dynamic range cannot be captured by scan).

#	Headline	Content
1	Title	The title should clearly and briefly summarise the topic of the SOP
2	Department, Date, ID	Department, date, and ID are information that must be recorded regarding the affiliation
3	Purpose	Here is a summary of the reason why there is an SOP in this context
4	Scope	The scope describes to whom the SOP applies and the reason why this is carried out
5	Definitions	All terms used in the SOP are defined to avoid misunderstandings

6	Procedure	The actual procedure to be followed

Table S1. This Table describes the general content of a standard operating procedure documentation.

3.2. Concrete example

Procedure DM-1: Assignment

In this example, the head of the IDIR first appoints a person responsible for documentation, who acts as a document representative.

Since the document representative cannot take over solely the documentation, further responsibilities of the documentation still need to be clarified. While each individual and projects are responsible for documenting their work and adhering to the policies, random checks can be made to ensure that they are carried out correctly within the guidelines of the QMS.

Procedure DM-2a: Documentation platform

The institute has chosen Apache's Subversion software as its documentation platform (Collins-Sussman, 2002). This platform meets the majority of the requirements, namely:

- Versioning
- Editing and access only by authorized users
- Renaming and deleting documents is versioned, thereby preventing data loss.

To check if all the necessary documents have been read by everyone, the institute has set up an email address. Whenever an employee has read a document, he/she/* sends a confirmation to this email address. These documents are managed and stored by the documentation representative.

To maintain structure, documentation within subversion is divided into categories. Categories like QMS, processes, and other global topics such as sales should be included as a minimum and these categories should be expandable at any time. In these categories, related documents can then be stored in a structured manner. A separate documentation folder is created for each project to store related documents and create an easily navigable structure.

Procedure DM-2b: Documentation platform (Code)

Since the code management is project-related, IDIR allows the projects to choose which code management system to use. The “main responsible person” for the project makes this decision; the decision must be documented in the “software

development plan” (see procedure PE-1) However, IDIR recommends its staff members to use Git to manage the code (Spinellis, 2012). By using Git, the properties required for code management are automatically maintained. There is also versioning and the possibility to store intermediate states in a referenceable way. Furthermore, it prevents the loss of code and enables developers to simultaneously work on the same code. Any decision not to use Git should be documented in the “software development plan”.

Procedure DM-3: Documentation lifecycle

The monitoring of the lifecycle of the documents is performed by the IDIR document representative.

While the creation of project-related documents is the responsibility of the project leader, the following procedure applies to global documents.

First, each employee can request that documents be created or modified. This request is then checked by the document representative and the task of creation is delegated. After the document has been created, it must be approved by persons who have the authority to accept it. In the IDIR, these are the leading positions and the document representative. These are versioned according to the regulations and released for reading by the employees. Old versions are marked as such and are then are no longer accessible.

To guarantee consistent storage, backups are required, which are performed by the IT department at weekly intervals.

Procedure DM-4: Documentation change

The procedure of “DM-3: Documentation lifecycle” must also be followed for changes. Furthermore, it is necessary to validate which documents are affected by the change and adjust them if necessary.

Procedure DM-5: Meeting minutes

In general, the person who sets up a meeting is responsible for its documentation. However, this can be delegated before the meeting. In the documentation of the meetings, there is also the possibility to implement alternative documentation for project-related meetings, as long as they comply with the standards defined in the QMS. For all meetings that take place project-independently, the following procedure is required.

Since the person responsible for documentation cannot be present at every meeting, the taking of protocols will be passed on in the group attending the meeting. Once all

the participants have taken the notes, they start again from the beginning. At one-time meetings, someone is needed to take the notes voluntarily, and if no one volunteers, someone is appointed by the chairperson of the meeting.

This process can be adjusted within a project for alternative meeting protocol requirements. All adjustments should be documented.

Procedure DM-6: Standard operating procedure documentation

SOPs can be described as guidelines on how certain applications should be carried out. An SOP is always structured similarly. To illustrate this with a practical example, an SOP for meeting protocolling is shown below.

#	Headline	Content
1	Title	Standard operating procedures for the recording of meetings
2	Department, Date, ID	Institute for Diagnostic Image Processing in Radiology, 24. Apr 2020, SOP-74922947
3	Purpose	To provide a procedure to define the general guidelines to ensure the recording of meetings is done correctly
4	Scope	This procedure applies to all meetings held independently of projects and is recommended for each meeting protocol.
5	Definitions	ID - Identification<br>Document representative - Working title for the person responsible for the documentation
6	Procedure	First of all, a recorder is selected. If the document representative is not present, the procedure is as defined in the QMS.<br>The recorder will first note the name of the department, the date, the meeting ID, and the project name to which the meeting belongs.<br>Furthermore, all attendees should be recorded by name.<br>Now the content of the meeting should be recorded

		in key points.<br>Afterward, the protocol is sent to all participants and stored in the documentation platform

Table S2. This Table illustrates an example for the definition of a standard operating procedure for the recording of meetings in the IDIPR institute.

4. Project planning

The IDIR handles projects in a broad range of sizes, ranging from a few months for a single person to multi-year with an entire team. The requirements the QMS put up must therefore find the balance of being so flexible that they fit all these different projects and on the other hand so precise that it does not void the benefits of having a QMS.

Procedure PP-1: Assignment

The QMS requires every project to fill out a “project start” form asking for project details. The specific “project start” form is stored in an SOP document which may change over time. However, the following fields are mandatory to fill – independent of the project size.

#	Item	Comment
1	Project name	A unique name helps to make sure everyone is talking about the same project
2	Main responsible person	Could be a “project manager”
3	Project Goal	High-level goal
4	Planned start and end date	
5	Success/termination criteria	Could be the finalized development of a “product”, the end of an available time window, certain funding used up, …
6	Planned resources	How many people for which time; any specific devices, suppliers, …

7	Any exclusions from procedures the QMS asks for – with reason	This is the place where the QMS can be tailored in a planned and systematic way

Table S3. This Table illustrates an example for the definition of a standard operating procedure document for project start in the IDIPR institute.

The filled-out "project start" form needs to be signed by the "main responsible person" and the head of the institute.

The head of the institute is responsible for this procedure. Responsibility will probably be delegated to the person who will be the "main responsible person", but at the time this process needs to be initiated, there is no assignment to this role yet. The assignment of the "main responsible person" role to a physical person is one of the results of this procedure.

Procedure PP-2: Prepare document storage

As soon as the project has a name, a folder with this name is created in the institute's main "projects" file system. All documents and records that will be created for this project must be stored in this folder or subfolders. A set of subfolders (one level deep) is defined in an SOP and will be created for each project. (Note: this includes subfolders like "Administration", "Testing", "Requirements", …). Further substructures may be defined by the projects as needed. Note: The scanned "project start" form is one of the documents to be stored here. The "main responsible person" is responsible for this procedure, which must be completed within 2 weeks after the assignment.

Procedure PP-3: List of documents

The next procedure that the QMS defines is the creation of a list of documents that will be created during the lifetime of the project, together with a responsible person for each of these documents. The purpose of this list is to make sure that no document is forgotten and it is clear who takes care of which document. The list of documents may be updated during the project, items may be added or removed, and no management approval is needed for an update, but for each change, the affected responsible persons must approve and a reason for the change must be documented. The "main responsible person" must create the initial version of this list within 4 weeks of the assignment.

Typical documents that would be included in this list would be the software development plan (see below), the requirements specification, the user manual, or a team member list.

5. Project execution

Again, due to the wide range of possible project sizes and types, the processes that the QMS defines need to find the right balance between flexibility and strict guidance. Projects can customize the procedures to a wide extent, but every customization must be documented.

Procedure PE-1: Create a software development plan

One document that each project must create is a software development plan. This document is the most fundamental document for the technical planning of the project. It lies within the responsibility of the “main responsible person” that this document is created and maintained. A template for this software development plan is again stored in an SOP document that may be adapted to needs over time; however, the following points must be addressed in each software development plan:

#	Item	Comment
1	Choice of the development process	E.g. Waterfall, Scrum, Kanban, any custom approach or none at all
2	Description of the development environment	Should be detailed enough to allow to re-create the development environment
3	Description of build and integration steps	Should be detailed enough to allow to re-create the final software
4	Description of requirements handling	Where do requirements come from, how are they prioritized, how are conflicting requirements handled, [how] are requirements documented
5	Plan for testing	High level, what level and what type of testing is planned (if no structured testing is planned at all, there is a reason for this – document it here)
6	Handling of changes	How will the project deal with changes during the development

Table S4. This Table illustrates an example for the definition of a standard operating procedure documentation for the software development plan in the IDIPR institute.

This may seem like quite a lot of writing for a small project, but the individual items may be very short for such a project. A single person working on a bachelor thesis would probably choose not to use a formal development process. This person would document the requirements as part of the thesis, perform only punctual functional tests, and decide whether or not to consider a change. All these steps would be compatible with the QMS. Describing the development environment and the development and integration process however is important even in such a scenario. Otherwise, the knowledge transfer of the results might be very difficult.

The IDIR considers it an important benefit that following the QMS procedures implies such decisions (e.g., to minimize the efforts for an item) are taken consciously and are documented so that it can be understood afterward why such decisions were made. This makes it much easier to retrospectively understand projects, helping any technology transfer, reproducibility, and reusability, significantly.

6. Management

To make sure the QMS system is being used, the IDIR needs to make sure every staff member is trained in QMS. Therefore, the following mandatory processes are defined:

Procedure MA-1: Assign quality management responsibility

One person in the institute is assigned the role of a quality management assignee. This person is the “owner” of the quality management documents and needs to approve any change to the QMS. Note: this approval is mainly to make sure that the quality management representative is aware of all changes and to avoid contradictions within the QMS. The head of the institute is responsible to make sure that the role is always staffed.

Procedure MA-2: Implement training for new staff members

All new staff members need to read and understand the QMS as it is at the time they start working at the institute. A small test is conducted afterward, with the main intent that people read the QMS. The supervisor of the new team member is responsible for the implementation.

Procedure MA-3: Implement department meeting with QM update

Additionally, there is a short update session at the beginning of each semester in which the entire institute gets an update on any changes to the QMS and can provide feedback or improvement suggestions. The head of the institute is responsible for the implementation but may delegate this to the quality management assignee.

Procedure MA-4: Annual QM review

The IDIR at this time chooses not to implement any specific measures to enforce the QMS, it is optimistic enough to assume the procedures will be followed voluntarily. An annual QMS review is planned in which this policy and the effectiveness of the QMS will be reconsidered. The head of the institute is responsible for organizing this meeting.

7. References

Collins-Sussman, B. (2002). *The subversion project: buiding a better CVS*.

https://dl.acm.org/ft_gateway.cfm?id=513042&type=html

Spinellis, D. (2012). Git. *IEEE Software*, *29*(3), 100–101.
